# On Growth and Morphology of TiO_2_ Nanotubes on CP-Ti by Anodic Oxidation in Ethylene Glycol Electrolyte: Influence of Electrolyte Aging and Anodization Parameters

**DOI:** 10.3390/ma15093338

**Published:** 2022-05-06

**Authors:** Bruno Ribeiro, Ruben Offoiach, Stefano Rossetti, Elisa Salatin, Maria Lekka, Lorenzo Fedrizzi

**Affiliations:** 1 Lima Corporate, Via Nazionale 52, 33038 San Daniele del Friuli, Italy; elisa.salatin@limacorporate.com; 2 Polytechnic Department of Engineering and Architecture, University of Udine, Via Cotonificio 108, 33100 Udine, Italy; ruben.offoiach@uniud.it (R.O.); stefano.rossetti@uniud.it (S.R.); lorenzo.fedrizzi@uniud.it (L.F.); 3 CIDETEC, Basque Research and Technology Alliance (BRTA), Po. Miramón 196, 20014 Donostia-San Sebastián, Spain; mlekka@cidetec.es

**Keywords:** TiO_2_ nanotubes, CP-Ti, grade 2 Ti, anodization mechanism, growth kinetics, ethylene glycol electrolyte, applied potential difference, voltage, time, electrolyte aging

## Abstract

Anodic oxidation of CP-Ti, for production of TiO_2_ nanotubes, has been extensively described in terms of the electrochemical mechanism of tubular growth or the effect of the parameters on the final tube morphology. Recently, a kinetic growth model was proposed to describe the distinct morphologies of the anodic oxide layer as phases of the nanotubular development process, offering a new perspective for the tuning of nanotube production. In this work, the anodizing behavior of a CP-Ti alloy in an ethylene glycol electrolyte was investigated in light of this new model. The final morphology of the nanotubes was characterized by SEM, considering the effects of electrolyte aging, the microstructure, the applied potential difference and time on the morphological development of nanotubes. Electrolyte aging was shown to lead to a decreased dissolution effect on the oxide. The applied potential difference was shown to lead to an increased dissolution effect and more rapid nanotube growth kinetics, while time resulted in extended dissolution. Moreover, the obtained results were analyzed considering a previous study focused on the anodizing behavior of the α- and β-phases of Ti6Al4V alloy. Overall, the tube morphology resembled that obtained for the Al-containing α-phase of the Ti6Al4V alloy, but the growth kinetics were considerably slower on CP-Ti.

## 1. Introduction

Over the last two decades, TiO_2_ nanotubes (TNTs) have been the focus of research for a large variety of applications, including solar cells, sensors, Li-ion batteries and medical implants. Such interest can be ascribed to their unique features, namely their electrical, optical and photocatalytic properties, high aspect ratio and bioactivity [1,2,3,4,5,6,7]. The self-organized growth of vertically-oriented TNTs on the surface of Ti alloys, by electrochemical anodization, was first achieved in 1999 by Zwilling et al. in fluoride containing electrolytes [8,9]. Due to its low cost, ability to produce vertically oriented nanotube arrays directly on the surface of the substrate, and good reproducibility, this method has been extensively researched by different groups in the following decades in order to optimize TNTs synthesis [2]. As a result, the influence of various electrochemical parameters on nanotube growth and morphology, namely the applied current or potential difference, as well as the respective ramp, time, temperature, stirring conditions, double-step anodization approaches, and electrolyte compositions have been extensively characterized [2,3,4,5].

Regarding solely the electrolyte composition, four synthesis generations of TNTs have been defined [1,4,10]. A first generation was grown in aqueous HF electrolytes, with low pH and high aggressivity towards both the Ti alloy and the anodic TiO_2_. The resulting nanotube arrays presented low organization, a length of only a few microns, and high side wall inhomogeneity. The replacement of HF by fluoride salts allowed for a higher pH and marked the second synthesis generation, resulting in nanotubular layers with a thickness several microns higher. The third generation was later achieved by replacing water-based electrolytes (generally known as aqueous electrolytes) with electrolytes based on organic solvents (generally known as organic electrolytes), which contain small amounts of fluoride ions (0.1–0.5 wt.%) and water (0.1–5 wt.%) [6,10]. Different organic solvents have been investigated, such as glycerol, formamide, ethylene glycol, dimethyl sulfoxide (DMSO), and N-methylformamide. The resulting nanotubular layers possessed, generally, a well-defined honeycomb-like structure on the top, smooth tubular walls, and very long lengths (up to 1000 µm in thickness) [5,6,11,12,13,14,15,16]. The fourth synthesis generation is outside the scope of this work, and therefore will not be addressed here.

These works have allowed to ascertain that the F^−^ ions in the electrolyte react with either Ti^4+^ ions or TiO_2,_ forming water-soluble fluoro-complexes ${\left[{\mathrm{TiF}}_{6}\right]}^{2-}$. On the other hand, water is both a supplier of oxygen for the formation of the metal oxide, and a solvent for ${\left[{\mathrm{TiF}}_{6}\right]}^{2-}$. Thus, self-organized nanotube growth begins with pore nucleation on the initially formed compact oxide, and is achieved through a dynamic equilibrium between metal/oxide dissolution and oxide formation that, in time, is established. Factors such as the pH, which depends on different supporting reagents, or the type of solvent, define this dynamic equilibrium in a way that ultimately governs the final morphology of the nanotubular layer [2]. For instance, the low water content in organic electrolytes has been shown to be a crucial factor for higher tubular lengths, since it is essential for oxide formation but mitigates the aggressiveness of the electrolyte [11]. Moreover, in these electrolytes, it is often observed that a “compact porous layer” is present at the top of the tubes. This layer is what remains of the compact oxide that is formed in the early stages of the anodization process. Its slower rate of dissolution in organic electrolytes allows for an extended period of tubular growth under it, protecting the forming tubes from dissolution by the electrolyte. This has been demonstrated by several works, such as the ones of Ali et al. and Albu et al., which used this effect in order to obtain well-defined and highly ordered TNT layers [12,13]. It is important to note that this layer is often identified by different names in different works. For instance, Ali et al. identified this layer as the “top nanoporous layer”, while in a previous work, our group identified it as the “porous compact oxide layer” [12,14]. In a recent comment by Mazare, which addressed the misidentification of this layer as nanopores in several published works, the author refers to it as the “Initiation Layer”, a term first used by Albu et al. [13,15] In this work, for a better understanding, this layer will be identified by the same term.

A further optimization step involves electrolyte aging, which is often performed in ethylene glycol-based organic electrolytes. This step has been deemed essential in order to achieve a reproducible and well-defined nanotubular morphology, with open tube tops. Moreover, it has been shown to enhance the mechanical stability of the produced TNT layer with a lower risk of delamination from the substrate. Aging involves the reutilization of the electrolyte in several anodization processes employing dummy Ti samples, before the target samples are prepared. Generally, this results in a decrease in the F^−^ concentration and the accumulation of TiF_6_^2−^ in the electrolyte. If the process is performed in a glove box, in controlled conditions without air moisture, the water content of the electrolyte decreases with each anodization. On the other hand, if it is performed in an air atmosphere, due to hygroscopic nature of ethylene glycol, it increases until saturation. Both the increase in the water content and the increase in the concentration of ${\left[{\mathrm{TiF}}_{6}\right]}^{2-}$ lead to an increase in the conductivity of the electrolyte [1,10,11,16,17,18].

Recently, a new kinetic model for nanotube growth was proposed by Seçkin and Ürgen, while studying the anodization process of pure Ti foils in an ethylene glycol-based electrolyte containing NH_4_F (0.3–0.6 wt%) and H_2_O (1 vol%). This model divides the growth process into three stages, defined by distinct growth kinetics, based on the evolution of the morphology of the anodic oxide layer. *Stage 1* includes the formation of the initial barrier oxide, pore nucleation, and nanotube growth under the slowly dissolving initiation layer, which has a protective role against dissolution exerted by the electrolyte. A very short *Stage 2* is reached upon the complete dissolution of that initial barrier oxide, and is characterized by visible, well-defined tubular structures. *Stage 3* occurs immediately afterwards, in which the extended anodization leads to the progressive formation of nanograss, due to the excessive dissolution of the tube tops [19].

Typically, the growth of TNTs has been characterized using the current–time transient curve, which illustrates the evolution of the current density with the anodization time. In this model, the initial sharp decrease of the current density has been attributed to the formation of the initial compact oxide; pore formation has been correlated with a moderate increase in current, and lastly, the quasi-steady-state values of the current have been correlated with the growth of TNTs under dynamic equilibrium. Thus, although both a compact oxide over the top of growing tubes and nanograss are well-known morphologies, with the latter being attributed to excessive dissolution by the electrolyte, they were often considered to be different resulting morphologies which could only be altered by changing the parameters of the anodization process, such as the electrolyte composition, time or voltage, or through post-anodization treatments [1,4,20,21]. As stated by Seçkin and Ürgen [19], the growth kinectics is usually not taken into account for the optimization of the process and control of the final morphology of the tubes.

In a previous work, our group investigated the influence of the alloy composition and microstructure on the growth mechanism and morphology of TiO_2_ nanotubes on Ti6Al4V, also known as grade 5 Ti (according to the ASTM standard classification for titanium and titanium alloys). In this study, an ethylene glycol-based electrolyte, containing ammonium fluoride, was used. It was demonstrated that the α + β dual-phase microstructure of the alloy resulted in a non-uniform anodic oxide. Over the α-phase, well-defined honeycomb-like nanotubes with thin tube walls were grown. Over the β-phase, the tubular structures had much thicker walls, smaller inner diameters, and lower heights. This was attributed, considering both the current–transient curve and the model proposed by Seçkin and Ürgen [19], to the different susceptibilities of the alloy phases to dissolution in the electrolyte [14].

In this work, the aim is to provide a study of the TNTs’ growth kinetics, through the anodic oxidation of commercially pure titanium (CP-Ti, referring to ASTM grade 2 Ti). For that purpose, the effect of electrolyte aging, applied voltage, time, alloy composition and microstructure (described by EDXS) on the nanotube morphology was investigated by field emission scanning electron microscopy (FESEM). The evolution of the oxide and its growth kinectics were determined by analyzing the different obtained morphologies, considering the growth model described by Seçkin and Ürgen [19]. On the other hand, the effect of the substrate composition was analyzed considering the previously obtained results on nanotube growth kinetics in Ti6Al4V alloy. For that purpose, the electrochemical anodization process conducted in this work was performed under similar conditions to the ones described in that previous study [14]. The production of TNTs through anodic oxidation on CP-Ti has been extensively described in the literature. The effect of parameters such as the applied voltage on the final tube morphology diameter and length has been thoroughly characterized. Their influence as well as different approaches, steps and procedures have been specified in order to obtain specific nanotube morphologies [2,3,4,5]. Nevertheless, a thorough understanding of the effect of these parameters on tube development and growth kinetics, in organic electrolytes has not yet been properly described, but it is important for commercial applications.

## 2. Materials and Methods

### 2.1. CP-Ti Specimen Preparation

**Figure 1:**
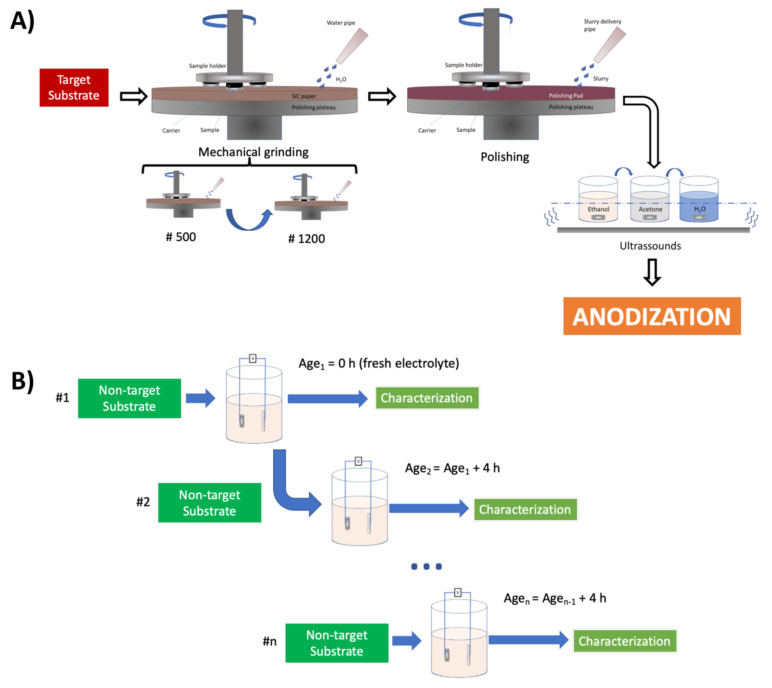
Schematic representation of (**A**) the process of specimen preparation prior to anodization and (**B**) electrolyte aging (#n: number n of anodizations performed in the same electrolyte).

Commercially pure Ti disks (CP-Ti; ASTM F67 alloy; grade 2) with a thickness of 4 mm and a diameter of 30 mm were used as substrates. All of the disks were cut from a rod of medical-grade CP-Ti provided by the Titanium International Group, SRL, Bologna, Italy. The chemical composition (wt.%) was 0.052 Fe, 0.14 O, 0.005 C, 0.003 N, 0.0009 H and Ti (balance). All of the substrates were ground in SiC papers (grades 500 and 1200), and later mechanically polished using a silica suspension (OP-S NonDry silica suspension 0.25, STRUERS with 25% H_2_O_2_). Then, the substrates were ultrasonically cleaned, sequentially, in ethanol, acetone and DI water baths, for 5 min each, and dried by air blowing. A schematic representation of this process is presented in Figure 1A. For the metallographic investigations, polished samples were chemically etched for 30 s using Kroll’s etchant (2% *v*/*v* HNO_3_ (68%), 1% *v*/*v* HF (48%)).

### 2.2. Electrolytic Anodization Process

TiO_2_ nanotubes were obtained through electrochemical anodization using the experimental setup previously described in [14], but with CP-Ti disks as the anode. In this study, nanotube layers were produced at various anodization times (1,3,6 and 9 h) and voltages (20, 40, 60 and 80 V) in an ethylene-glycol electrolyte containing ammonium fluoride (EG + NH_4_F), in order to study the effect of these parameters on the nanotube layer morphology. Prior to these tests, and in order to study the effect of electrolyte aging on the nanotube morphology and process reproducibility, different batches of the electrolyte were used in repeated anodization steps at 40 V for 4 h of dummy samples. In each step, the samples were replaced. A schematic representation of this process is represented in Figure 1B. All of the samples, after anodization, were cleaned by ultrasonication in acetone for 3 min and air dried.

### 2.3. Surface Analysis and Morphology Characterization

A field-emission electron microscope (JEOL model JSM-7610FPlus (Tokyo, Japan) equipped with an Oxford X-MAX20 energy dispersive X-ray spectrometer (EDXS) (Oxford Instruments, Abingdon, UK)) was used to assess the microstructure and chemical composition (determined by EDXS) of the CP-Ti alloy, as well as the morphology of the anodized specimens. Micrographs were captured in the middle region of the anodized area using a secondary electron in-lens detector. For the analysis of the specimens in cross-section, micrographs were taken of samples with a mechanically detached oxide, in a small section of their surface.

## 3. Results

### 3.1. Microstructural Analysis of the Base Alloy

**Figure 2:**
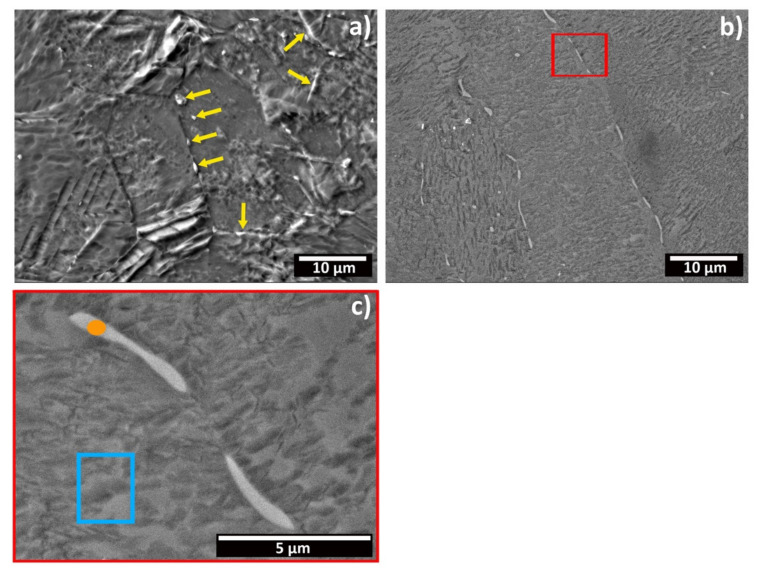
FESEM micrographs of the bare CP-Ti sample: (**a**) SE micrograph of a sample etched in Kroll’s solution for 30 s, (**b**) backscattered electrons of a polished sample, and (**c**) detail of figure (**b**) (red frame).

Figure 2 shows FESEM micrographs of polished CP-Ti samples, either after metallographic etching in Kroll’s solution for 30 s (Figure 2a) or without etching in backscattered mode (Figure 2b,c). The samples present equiassic, elongated grains of sizes varying between 15 to 50 µm. In addition, some intermetallic grains are also identified, aligned along the grain boundaries (yellow arrows Figure 2a and red frame Figure 2b). The EDXS analysis conducted on the polished samples (Table 1), in the regions highlighted in orange and blue (Figure 2c), shows that these intermetallic particles present higher concentrations of Fe, Cr and Ni, which are known beta stabilizers. As such, these grains can be classified as small intermetallic β-phase grains.

The presence of these intermetallic grains on the microstructure of Ti alloys is well known [22,23]. Unalloyed Ti is categorized in four grades, according to the ASTM standard. The main distinction between them is the yield strength, which is determined by the specific amount of these intermetallic elements, including Fe [24]. The presence of Ni and Cr in the EDXS analysis (Table 1) was somewhat surprising, because it is not described in the standard, and is generally not reported. Nevertheless, it is important to highlight that Ni and Cr are known beta-phase stabilizers, and that the CP-Ti composition can include some residual amounts of other compounds [25]. Moreover, their amount was quite small and limited to these intermetallic particles, as revealed by the EDXS analysis. From our analysis, these intermetallic particles seem to be uniformly distributed across the surface of the samples, existing at grain boundaries and triple junctions, which is in accordance with the results reported by Tesař et al. [22].

### 3.2. Influence of Electrolyte Aging on the TiO_2_ Nanotube Morphology

**Figure 3:**
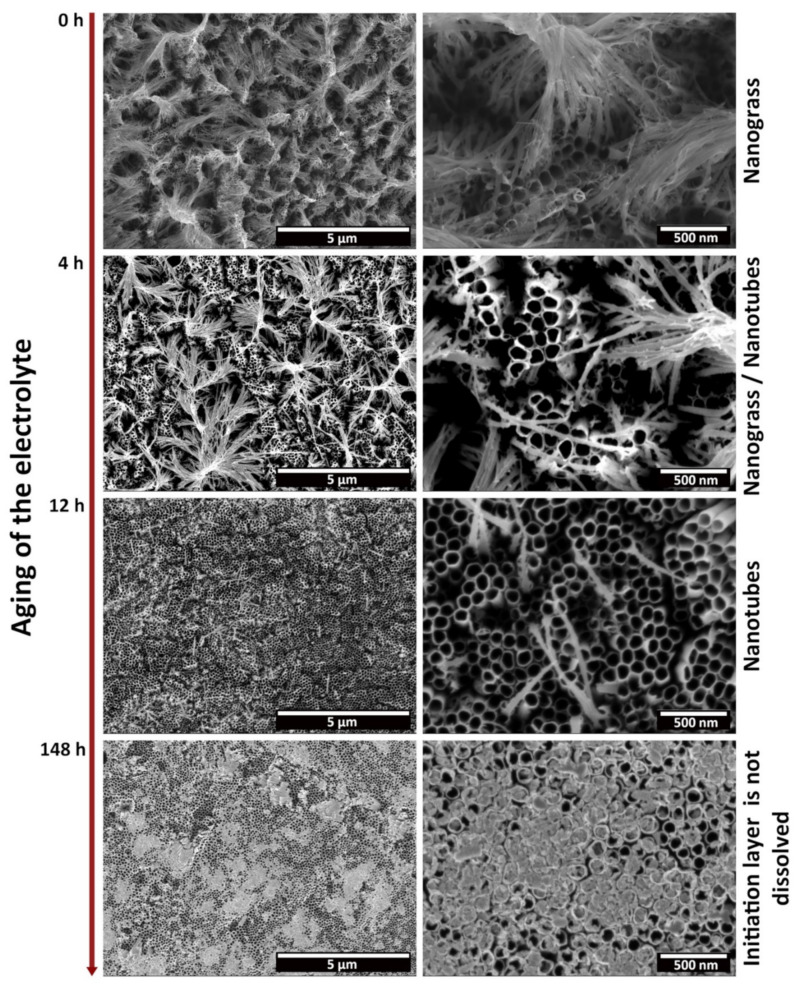
Morphology of the anodic oxide layer grown over CP-Ti, after anodization at 40 V for 4 h, with regard with the aging of the electrolyte. Aging is measured in terms of the accumulated hours of usage of the electrolyte, prior to the anodization of the sample.

Figure 3 shows FESEM micropgraphs depicting CP-Ti samples anodized at the same applied potential difference (40 V) and time (4 h), for an electrolyte with increasing usage. The specimens anodized using a fresh electrolyte (0 h of usage time) presented a great amount of nanograss on their surface. By increasing the age of the electrolyte, the nanograss morphology was gradually replaced by well-defined nanotubes with open tops and, if the aging was too extensive, by a partially-dissolved initiation layer. From these results, a prior electrolyte usage above 12 h but below 148 h was determined to be necessary in order for well-defined open-top nanotubular structures to be obtained in a reproducible way.

The effect of electrolyte aging has been extensively studied. In general, various works agree that the increasing usage of the electrolyte results in lower overall conductivity, a reduced concentration of F^−^ in solution, the accumulation of [TiF_6_]^2−^, and a reduced concentration of NH^4+^ ions due to the formation of (NH_4_)_2_TIF^6^ salt or volatile NH_3_ [17,18,26]. For this reason, some authors also report an increase in the pH of an EG + NH_4_F electrolyte, from a weak acid to an alkaline solution, due to formation of ammonia and the electrolysis of water [18,26]. However, not all of the authors agree on these effects. Lee et al., for instance, reported an increase in conductivity with electrolyte usage, but used an EG + HF electrolyte instead of NH_4_F, which could explain the distinct reported results. An acidic pH mainly induces the formation of TiF_2_^6−^ which is highly soluble in water, while a more alkaline pH could induce the formation of less-soluble oxofluorides, which remain in the TiO_2_ film [16]. Indeed, Suhaldonik et al. detected a lower amount of fluorides in the nanotubes produced after the prior usage of the electrolyte, which seems to corroborate this theory. Another point of divergence is the variation in the water content. Suhadolnik reported an increase in the water content, while Gulati et al. reported a decrease [17,18]. This could be a result of the different conditions in which the anodization processes were performed. If the process is carried in air atmosphere, the water concentration increases until saturation. If it is conducted in a controlled environment, without air humidity, such as a glove box, it decreases [17]. Ethylene glycol is a hygroscopic substance, and as such it absorbs moisture from the air, until saturation [10]. The results presented in this work support these findings.

Considering the model of Seçkin and Ürgen [19], the transition from a nanograss morphology (*Stage 3*) to, first, well-defined nanotubes with open tops (*Stage 2*), and later, to a partially dissolved initiation layer (*Stage 1*), points to a decrease in the dissolution capacity of the electrolyte with the increasing aging. It becomes clear that nanotube formation occurs at a slower rate. This could be attributed to the consumption of free F^−^ ions in the solution, and the increase in species such as [TiF_6_]^2−^ and (NH_4_)_2_TiF_6_, or oxofluorides which do not have a dissolution capacity against neither Ti or TiO_2_. Therefore, it can be assumed that the increasing usage of the electrolyte hinders nanotube formation, until they are not formed completely. Moreover, Guo et al. also reported on the increasing inter-tubular distance with the age of the electrolyte, but that could not be confirmed in this work [26]. In general, our studies have shown that an aged electrolyte, with 12 to 148 h of prior utilization, resulted in higher tube morphology definition at specific voltages for CP-Ti. For comparison, in our previous work on grade 5 Ti [14], it was determined that an anodization time of between 5 h and 20 h resulted in well-defined morphologies.

**Figure 4:**
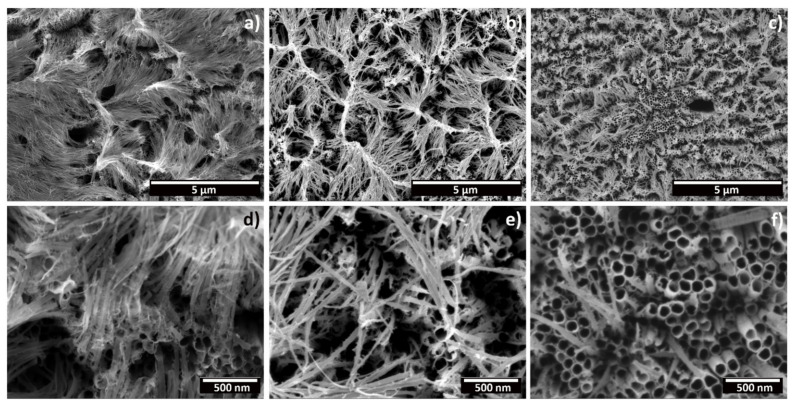
FESEM micrographs depicting a top view of the anodic oxide layer produced through electrochemical anodization in a CP-Ti substrate, without a post-anodizing cleaning step in an ultrasounds bath. (**a**,**d**) Low- and high-magnification micrographs of samples anodized in a fresh electrolyte (0 h of prior usage); (**b**,**e**) low- and high-magnification micrographs of samples anodized in an electrolyte with 6 h of prior usage; (**c**,**f**) low- and high-magnification micrographs of samples anodized in an electrolyte with 12 h of prior usage. All of the depicted samples were anodized at 40 V for 4 h.

Nevertheless, it is important to mention that the application of an ultrasound cleaning step, after anodization, could have an effect on the morphology of the oxide layer. Ultrassonic agitation, post-anodization, has been reported to lead to the partial remotion of nanotubes from the substrate [12]. Furthermore, it is also considered to be an optimizing step to efficiently remove superficial nanograss in order to obtain well-defined nanotubes [27]. For this reason, in Figure 4 we report the top-view morphology of the samples produced with fresh electrolyte (0 h), a 6 h aged electrolyte, and an electrolyte aged above 12 h without a cleaning step in an ultrasounds bath. Specifically for this analysis, the samples were soaked in acetone and air dried. As it can be seen, for all three samples, the amount of nanograss present is higher. Therefore, the synergistic role of ultrasounds in post-anodizing cleaning steps, regarding the resulting tubular morphology and reproducibility of the process, needs to be taken into consideration when analyzing the obtained morphology of produced TNTs.

### 3.3. Morphology of the TiO_2_ Nanotubes

**Figure 5:**
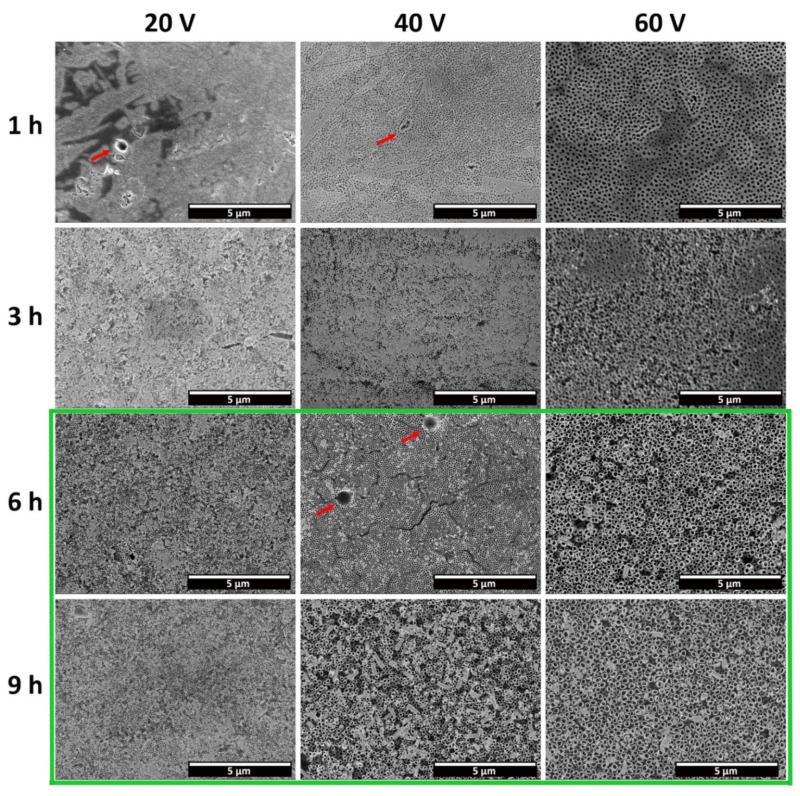
FESEM micrographs depicting a top view of the anodic oxide layer produced through electrochemical anodization in a CP-Ti substrate, at different applied potentials and times. The green frame highlights the conditions in which open-top nanotubes can be distinguished. The red arrows indicate cavities present on the oxide.

Figure 5 shows FESEM micrographs depicting a top view of CP-Ti samples anodized using different applied potentials and times, at low magnifications. Based on the results reported in Section 3.2, the electrolyte used to produce these samples had between 12 h and 148 h of prior usage time. Moreover, all of the samples were produced with a post-anodization cleaning step with ultrasound. From these micrographs, it is possible to observe that a compact porous oxide, which covers the entirety of the analyzed area, was obtained for all of the samples anodized for 1 h. The samples anodized for 3 h seem to present a compact oxide that is partially dissolving. This is especially visible in the micrograph obtained for the sample anodized at 60 V, which presents some zones which are still covered with the porous compact oxide, while on others open-mouth nanotubes can be observed.

A similar morphology was also observed on the samples anodized at 20 V for 6 h, but in this case the area with compact porous oxide was much smaller. For all of the other conditions, namely 40 V and 60 V for 6 h and 9 h, and 20 V for 9 h, open and well-defined TNTs were obtained. However, the nanotubes produced at 40 V and 60 V for 9 h were partially fragmented. This could be due to the high length of the TNTs produced after 9 h of anodization, which can cause their fragmentation during the post-anodization cleaning procedures with ultrasounds.

Compared to previous results on CP-Ti, it is worth mentioning the works of Durdu et al. [28], Regonini et al. [29], Sopha et al. [30] and Hilario et al. [31]. All of these works preformed the anodization of grade 2 Ti in electrolytes with similar compositions, using ammonium fluoride, but with higher amounts of water. Durdu et al. managed to form well-defined, open-top TNTs at a minimum voltage of 20 V for an anodization time of 1 h in electrolytes containing 5% V of DI H_2_O (compared to 2.5% V in this work). Regonini et al., in an electrolyte with 3.8% V of water, reported the over-dissolution of the tube tops at a voltage as low as 30 V, for 1 h of anodization. Hilario et al. and Sopha et al. managed to obtain well-defined, open-top nanotubes at an anodization time as low as 45 min, at 60 V, for an electrolyte with 7.5 % V and 10% V of water, respectively. Clearly, compared with the results reported in Figure 3, the increase of the water content in the electrolyte allowed for more rapid TNT growth kinetics.

This is further confirmed by the results obtained in aqueous electrolytes of the first or second synthesis generation, which have been reported since 2005. For instance, Vranceanu et al. reported that open-top nanotubes can be obtained by anodic oxidation in an aqueous HF electrolyte solution, at an applied potential difference of 20 V for 30 min [32]. Park et al. managed to obtain open-top, well-defined nanotubes at voltages as low as 1 V for 1 h of anodization. In this work, an aqueous HF electrolyte, supplemented with H_3_PO_4_, was used [33]. From this, it is clear that the higher the aggressiveness of the electrolyte, the faster the nanotube development. Similar conclusions have been reported by Sreekantan et al., who used electrolytes of the second synthesis generation but with varying pHs of 3, 5 and 7. The authors stated that the higher the pH of the electrolyte, the longer the anodization time for the initial formation of pits, and the lower the tube growth rate [34].

Regarding the anodization of the Ti6Al4V alloy [14], it is worth mentioning that well-defined nanotubes were obtained for an anodization time as low as 1 h (for 60 V) and applied potential differences as low as 40 V (for 2 h) in the same electrolyte composition, under optimized conditions of aging. For grade 2 Ti, open, well-defined nanotubes could only be obtained for anodization times above 6 h, at 20 V, 40 V or 60 V of applied potential difference. For lower anodization times, a compact, porous layer is present. This distinct behavior highlights the effect of the alloy composition on tube growth kinetics. As was thoroughly described for grade 5 Ti, the alloy composition will in turn influence the composition of the oxide, which ultimately influences the dissolution effect of the electrolyte and the kinetics of tube development [14]. As such, it should also be expected that unalloyed grade 2 Ti presents a distinct anodizing behavior than grade 5 Ti. These results, together with the ones obtained in Section 3.1, suggest that pure Ti is more stable than either the Ti-Al alloy or of Ti-V alloy of each phase of grade 5 Ti, and tube development occurs at a slower rate.

**Figure 6:**
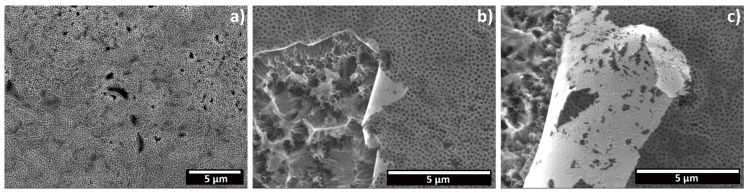
FESEM micrographs depicting a top view of the oxide layer produced on CP-Ti through electrochemical anodization at 80 V for 1 h: (**a**) view of the porous compact oxide; (**b**,**c**) detail of the detachment of the initiation layer, revealing the nanograss structure underneath.

A further increase of the applied potential to 80 V led to the formation of a particular morphology (Figure 6) in which the top surface is covered by the initiation layer (Figure 6a), and in some areas a delamination occurs, revealing a nanograss structure under it (Figure 6b,c). This result it is not in accordance with the model proposed by Seçkin and Ürgen [19], which considers that the formation of nanograss occurs due to the continuous dissolution of the tube walls only after the complete disappearance of the initiation layer which is a common view shared by other authors [10,35]. In any case, the thinning of the tube walls under the slowly dissolving initiation layer has been described by Albu et al., due to the etching action of the electrolyte [13]. As such, it is reasonable to consider that the anodization time and applied potential difference were not enough to completely dissolve the initiation layer but led to the rapid growth rate of the nanotubes underneath. Then, this resulted in an extensive thinning of the tube walls, which collapsed, forming nanograss. Finally, the initiation layer delaminated due to the loss of mechanical support.

Compared to grade 5 Ti [14], it is worth mentioning that well-defined TNTs were obtained for anodic oxidations conducted at 80 V for 1 h. From these results, it seems that nanotube growth and definition is more accelerated with voltage, while the oxide in CP-Ti is not so susceptible to dissolution by the Eg + NH_4_F electrolyte, ultimately resulting in different susceptibilities to both anodization time and voltage. Similarly, it is also interesting to note that in the work of Durdu et al. [28], well-defined nanotubes were obtained for anodization voltages as high as 100 V and 1 h of anodization time. This further confirms that morphology is determined by the susceptibility of the produced TiO_2_ to the dissolution exerted by the electrolyte.

**Figure 7:**
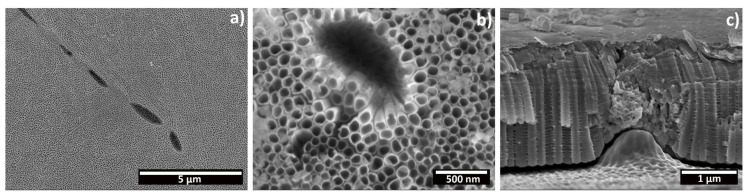
FESEM micrographs of the cavities in the anodic oxide: (**a**) top surface morphology at a low magnification, (**b**) detail of a cavity in the top morphology, and (**c**) a cross-section view.

Moreover, some cavities were noticed on the surface of all of the anodized samples, where the anodic oxide seems not to grow (red arrows, Figure 5). Micrographs reporting in detail this phenomenon, both on the top surface and in cross section, are shown in Figure 7. The shape, size and distribution of these defects closely resembles those of the β-phase intermetallic grains seen in Figure 2. EDXS analysis preformed over these cavities revealed the presence of Fe, confirming that they are formed over these grains. Micrographs in cross section (Figure 7c) revealed that well-defined nanotubes are produced over the equiassic α-phase grains, while a sponge-like, porous oxide seems to have been produced over the β-phase intermetallics. As such, the composition of the substrate influences the morphology of the produced oxide, and the presence of Fe, Cr or Ni as alloying elements hinders the formation of nanotubes over these regions.

**Figure 8:**
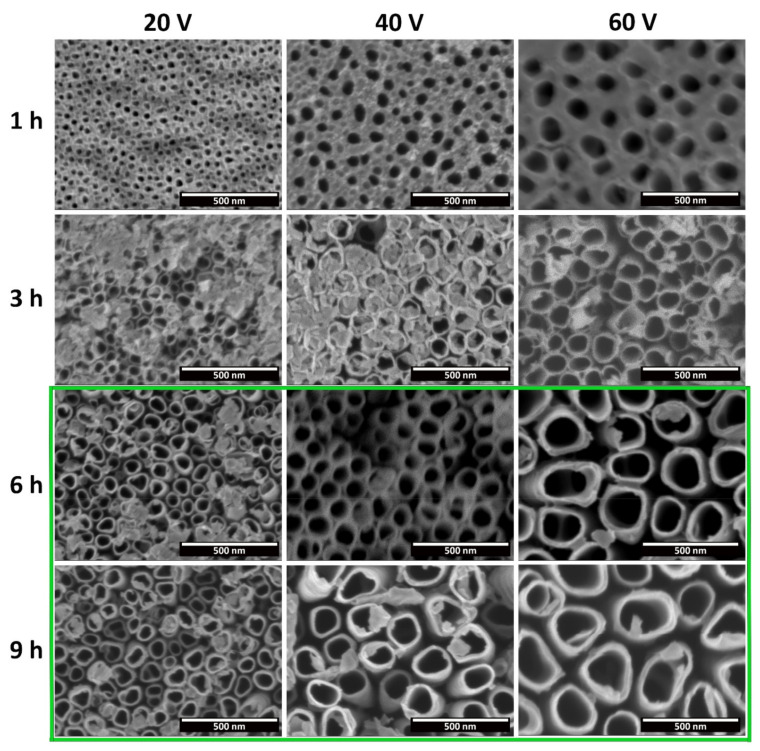
FESEM micrographs depicting a top view of the anodic oxide layer produced through electrochemical anodization in a CP-Ti substrate, at different applied potentials and times, at a higher magnification. The green frame highlights the conditions with open-top nanotubes.

Higher-magnification micrographs of the nanotubes’ top surfaces are reported in Figure 8. These micrographs confirm the reported results in Figure 5, and allow us to clearly distinguish the morphological differences between nanotubes produced under different applied potential differences and anodization times. Overall, the tube morphology closely resembles that obtained for the nanotubes grown over the Al-containing α-phase of Ti6Al4V alloy, as described in [14]. Moreover, and according to the model proposed by Seçkin and Ürgen [19], the obtained results seem to show both *Stage 1* (nanotubes with an initiation layer) and *Stage 2* (open-topped, well-defined nanotubes) of nanotubular development. Additionally, at 3 h of anodization time, it is possible to observe the transition between the two stages (the partially dissolved initiation layer).

The effect of the time can be easily perceived in the increased dissolution of the initiation layer. By increasing the time from 1 h to 9 h, at all of the applied potential differences, there is a transition from a compact barrier oxide (*Stage 1*) first to tubes covered with the remnants of that initiation layer (various degrees of transition from *Stage 1* to *Stage 2*) and then to well-defined, open nanotubes (*Stage 2*). A similar effect, albeit to a smaller degree, is observed with the increase in potential from 20 to 60 V, especially for anodization times superior to 1 h. However, as previously described in Figure 5, the increase in the applied potential to 80 V did not lead to open, well-defined nanotubes, but instead to the formation of nanograss under the not-yet-dissolved initiation layer. Moreover, the increase in the applied potential difference, at any given time, as well as the increase in time, for any given applied potential, leads to a marked increase in the nanotube diameter. Thus, these results show that both time and the applied voltage result in an increased dissolution of the anodic oxide, albeit through different mechanisms. The applied potential difference directly determines the strength of the electrical field, and thus the ion migration rat and the dissolution rate. On the other hand, longer anodiztion times result in an extended dissolution effect. Curiously, for grade 5 Ti [14], it is not possible to associate the dissolution effect preferably with either only voltage or time. However in this work, for the conditions tested, the variation in time was the one that more easily led to the transition in morphology.

**Figure 9:**
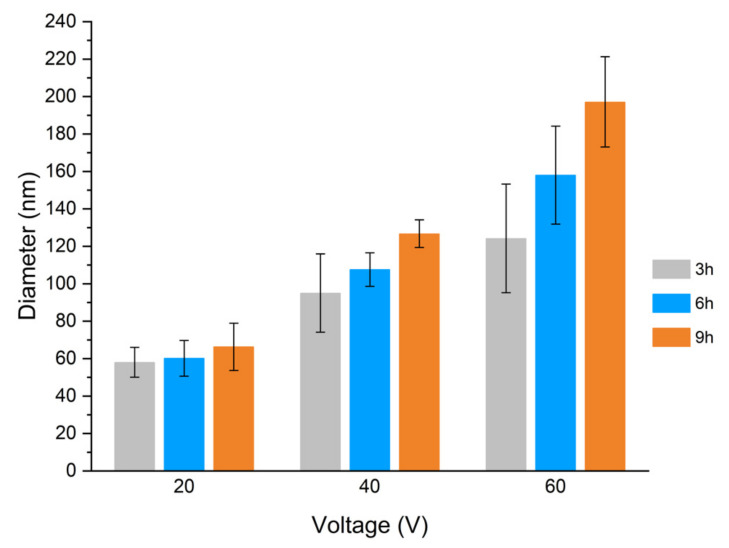
Average inner diameter of TiO_2_ nanotubes produced over CP-Ti as a function of the applied potential, for anodization times of 3 h, 6 h, and 9 h.

The average inner diameter of the tubes/pores is plotted in Figure 9. Overall, as was already noted in Figure 5 and Figure 8, these results show that there is an almost linear increase of the diameter with both the increase in the applied potential difference and the anodization time. For an applied potential difference of 20 V, the diameter was approximately 55 nm after 3 h, 60 nm after 6 h, and 67 nm after 9 h. Similarly, at 40 V the diameter was about 95 nm after 3 h, 108 nm after 6 h, and 127 nm after 9 h; at 60 V, the diameter was about 125 nm after 3 h, 158 nm after 6 h, and 197 nm after 9 h. These values show that the variation of the inner diameter of the tubes is more pronounced with the variation of the applied potential difference than with the variation of the time. However, with the increase of the applied potential, the effect of the anodization time on the tube diameter becomes more noticeable.

The pore diameter is closely related to pore nucleation during the initial stages of nanotube development. The applied potential difference will determine the strength of the applied electric field and, as such, the ion migration rate and the dissolution rate, for a specific electrolyte. Thus, the effect of the applied potential difference over the tube diameter is well-known, and similar results have been extensively reported by several authors for the anodization of CP-Ti in various electrolyte compositions, either aqueous or organic based [34,36,37,38]. The effect of increasing times, on the other hand, is usually associated with the increasing length of the tubular structures, as well as their morphological development from pores to tubes, or formation of nanograss due to over-dissolution. Regarding its influence over tube diameter, the effect has been investigated less, with some works presenting contradictory results. For instance, Chernozen et al. reported an increase in diameter when the anodization was extended from 30 min to 240 min, at 60 V (4 h) [39]. Bervian et al., on the other hand, reported a decrease in the diameter when increasing the anodization time from 60 to 240 min, similarly at 60 V [40]. However, these two works used distinct electrolyte compositions and counter electrodes. Chermozen et al. used an ethylene glycol electrolyte with approximately 1% V of Di H_2_O and 0.4 wt.% of NH_4_F, and another Ti foil as counter-electrode. On the other hand, Bervian et al. used a glycerol + ethylene glycol mixture with 0.25 wt.% NH_4_F and 2 wt.% H_2_O, and Pt as the counter electrode. These differences resulted in the different expected conductivity and overall kinetics of the reaction, and therefore it is not possible to draw a conclusion regarding the role of time on the tube diameter. In addition, it is worth mentioning that, in our previous work in grade 5 Ti [14], similar diameters were reached in much lower anodization times. For instance, for 60 min of anodization time, well-defined nanotubes were obtained with a diameter of 75 nm for 40 V, reaching 165 nm at 100 V (which for CP-Ti were obtained for anodizations superior to 6 h, at 40 V and 60 V, respectively). Moreover, while the diameter of the tubes increased with both time and applied voltage for grade 2 Ti, in the Ti6Al4V alloy, at 60 V, an anodization time of 120 min led to an inferior diameter compared to that obtained for nanotubes after anodic oxidation for 60 min. This inversion, as discussed in [14], could be attributed to an increased dissolutive action by the electrolyte.

**Figure 10:**
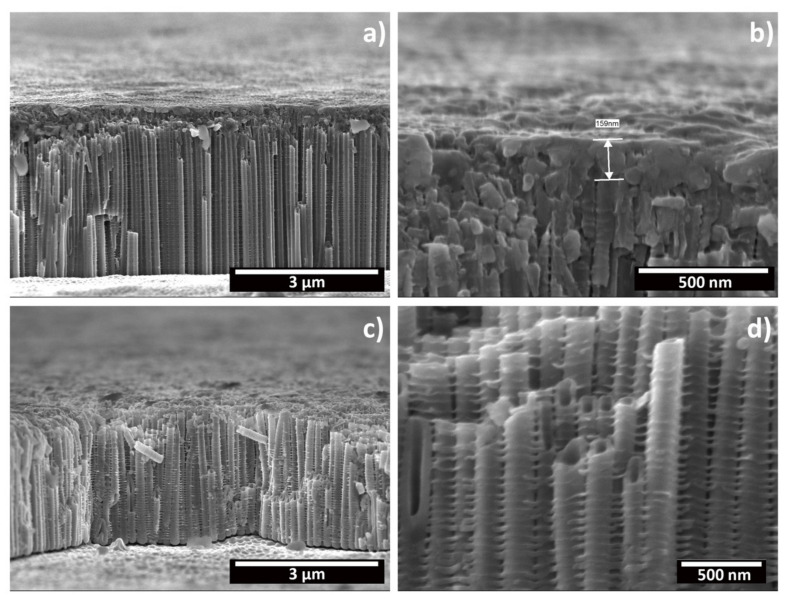
FESEM micrographs depicting the oxide layer produced on CP-Ti through electrochemical anodization at 40 V for 1 h (**a**,**b**) or 6 h (**b**,**d**) in cross section. (**a**,**c**) General view, (**d**) a detailed view of the initiation layer and tube tops, and (**d**) a detailed view of the tube side-wall morphology.

Figure 10 shows FESEM micrographs that depict a cross section of nanotubes produced at 40 V for 1 h and 6 h. For all of the samples, tubular structures present side wall inhomogeneity with ripples along the tube wall (Figure 10a,b). In Figure 10b, the tube tops are shown from a side view. For the 1 h sample, the initiation layer covering the tube tops is visible, with a thickness of 159 nm. However, no similar layer was observed for the samples produced at 6 h, which present completely open tube tops (see Figure 8). These results further confirm the dissolution effect of the anodization time. The presence of ribs along the tubular walls has commonly been described for nanotubes grown in aqueous electrolytes, but in organic electrolytes it is generally considered that the low water content prevents their formation, and only if the water content remains below a certain threshold. [10,11] Mechanistically, in aqueous electrolytes, their occurrence has been described to result in a temporary local acidification at the pore tips, which leads to a momentarily increased dissolution rate, resulting in a variation in the wall thickness of the forming tube [41,42]. In organic electrolytes, this has been ascribed to oxygen evolution due to electrolysis of water, which results in oxide rings that are later converted into ribs due to the chemical dissolution exerted by the electrolyte [42,43]. Additionally, the mechanical stresses and expansion factors, related to the growth kinetics and the applied electric field, are also considered to contribute to the formation of these ribs [21,44]. In particular, the work of Martinez et al., which compared the morphology of nanotubes produced on CP-Ti and on various binary Ti alloys, has shown that the morphology and quantity of ribs varied depending on the composition of the substrate [44]. Thus, the results obtained in this work suggest that the water content, under optimized conditions of electrolyte ageing, is above that minimum threshold for grade 2 Ti, which at 40 V for times of either 1 h or 6 h, resulted in the formation of these ribs. Curiously, under optimized conditions, smooth tube walls were obtained for the same electrolyte after an anodization at 60 V for 1 h, for Ti6Al4V alloy [14].

## 4. Conclusions

In this work, the growth kinetics of TiO_2_ nanotubes on grade 2 Ti, through anodic oxidation in an ethylene glycol electrolyte, using ammonium fluoride, were thoroughly characterized. Different potential differences, anodization times, and a study on the effect of electrolyte aging were applied for this purpose. The results show that the chemical composition of the substrate has a strong effect not only on the resulting final morphology of the anodic oxide but also on the growth kinetics of the nanotubes.

Overall, the nanotubes grown over the equiassic α-phase grains on CP-Ti present a similar morphology to the ones obtained on the Al-rich α-phase of the Ti6Al4V alloy in [14]. The oxide formed over Fe-rich intermetallic grains, on the other hand, did not possess a nanotubular structure, unlike the one grown over the V-rich β-phase of grade 5 Ti. In general, the kinetics of tube development were considerably slower in Cp-Ti than in Ti6Al4V. This has been shown by the effect of electrolyte aging and the anodization time. In order to obtain well-defined, open-top nanotubes, the required aging of the electrolyte was of a minimum 5 h for grade 5 Ti, compared to a minimum of 12 h for grade 2 Ti. Similarly, the anodization times (minimum 1 h vs. 6 h, respectively) were smaller on grade 5 Ti. Thus, the presence of Al as an alloying element seems to increase the kinetics of tube development for the same initial composition of the electrolyte used. Moreover, the α + β dual-phase microstructure of the grade 5 alloy may also have contributed to the difference in growth kinetics. In any case, it is clear that both the CP-Ti substrate and the oxide formed are much less susceptible to dissolution in the studied electrolyte, and as such, the tube growth kinetics are slower.

Lastly, it is important to mention that the specific susceptibility of the substrate in the electrolyte can also influence the effect on the morphology of specific parameters of the anodic oxidation process. For instance, on CP-Ti, the produced nanotubes present a more linear and clear variation of the diameter with both the increase in time and the applied voltage (while the effect of time was not so clear in Ti6Al4V alloy). Moreover, an anodization conducted at 80 V for 1 h led to the production of nanograss under a not-yet-dissolved inititation layer on CP-Ti (while open and well-defined nanotubes were obtained for the same conditions on Ti6Al4V alloy). In addition, the tubes produced on grade 2 Ti presented ripples along the walls, while the ones produced on grade 5 Ti had smooth walls.

## Figures and Tables

**Table 1:** EDXS analyses’ results in wt.% (Figure 2c).

Element		Blue	Orange
Ti	wt.%	96.07	86.1
O	wt.%	3.93	4.57
Cr	wt.%		0.71
Fe	wt.%		7.92
Ni	wt.%		0.7
Total	wt.%	100.0	100.0

## Data Availability

The data that supports the findings of this study are available from the corresponding author (B.R.) upon reasonable request.
